# Body Mass Index, Height, and Head and Neck Cancer Risk: The Japan Public Health Center-based Prospective Study

**DOI:** 10.2188/jea.JE20240033

**Published:** 2025-04-05

**Authors:** Seitaro Suzuki, Taiki Yamaji, Motoki Iwasaki, Manami Inoue, Shoichiro Tsugane, Tomohiro Shinozaki, Norie Sawada

**Affiliations:** 1 Division of Cohort Research, National Cancer Center Institute for Cancer Control, Tokyo, Japan; 2 Division of Epidemiology, National Cancer Center Institute for Cancer Control, Tokyo, Japan; 3 Division of Prevention, National Cancer Center Institute for Cancer Control, Tokyo, Japan; 4 International University of Health and Welfare Graduate School of Public Health, Tokyo, Japan; 5 Department of Information and Computer Technology, Faculty of Engineering, Tokyo University of Science, Tokyo, Japan

**Keywords:** head and neck cancer, oral and oropharyngeal cancer, body mass index, height

## Abstract

**Background:**

Although both a lower and a higher body mass index (BMI) are reportedly associated with head and neck cancer (HNC), reports from Asia are scarce. Moreover, evidence regarding the association between height and HNC is limited.

**Methods:**

We investigated associations between BMI, height, and the incidence of HNC among 102,668 participants (49,029 men and 53,639 women) aged 40–69 years in the Japan Public Health Center-based Prospective Study. We followed participants from 1990 to 2013. We conducted a Cox proportional hazards regression analysis, which included adjustment for potential confounders, such as smoking status. Baseline weight and height information were self-reported.

**Results:**

Over an average follow-up of 18.7 years, 311 HNC cases were newly diagnosed. Lower BMI was significantly associated with HNC, with hazard ratios (HR) of 2.75 (95% confidence interval [CI], 1.63–4.64) for <18.5 kg/m^2^ and 1.63 (95% CI, 1.15–2.30) for 18.5–20.9 kg/m^2^ compared to 23–24.9 kg/m^2^. Increased risk was suggested for higher BMI, with an HR of 1.30 (95% CI, 0.84–2.00) for ≥27.5 kg/m^2^. This trend was also observed in quadratic models. Results were similar among never-smokers. Meanwhile, only lower BMI showed a strong association with HNC risk among former and current smokers (HR 3.09; 95% CI, 1.54–6.20 for <18.5 kg/m^2^ compared to 23–24.9 kg/m^2^). Height showed no association with HNC.

**Conclusion:**

Lower BMI was significantly associated with HNC risk, while increased HNC risk was suggested in higher BMI among never-smokers. Among former and current smokers, only lower BMI was associated with HNC risk.

## INTRODUCTION

In Japan, estimated rates for the age-standardized incidence of head and neck cancer (HNC) by sex were 12.42 per 100,000 men and 3.71 per 100,000 women in 2015, and an upward trend in incidence has been observed.^1^ Interestingly, although increased body mass index (BMI) is associated with several cancers, such as oesophageal adenocarcinoma and renal cancer, lower BMI is associated with lung cancer risk.^2^ Regarding the Japanese population, a U-shaped association has been reported for BMI with total cancer risk in men.^3^ To date, however, associations between lower BMI and risk of HNC in Japan have not been investigated.

The World Cancer Research Fund (WCRF) has reported that overweight or obesity raises HNC risk.^4^ Meanwhile, the International Head and Neck Cancer Epidemiology Consortium (INHANCE) has reported that leanness, not obesity, shows an association with increased HNC risk, regardless of smoking and alcohol consumption.^5^ Furthermore, BMI ≥30 kg/m^2^ shows an association with the increased HNC risk in never-smokers, even though a pooled analysis of 20 cohorts reported that BMI <21 kg/m^2^ also showed an association with HNC risk among current smokers.^6^ The association of BMI with risk of HNC may, therefore, differ in accordance with smoking status.

In conjunction with BMI, height is another anthropometric measure that has been linked to cancer risk.^7^^–^^9^ INHANCE reported that height shows an inverse association with HNC risk.^10^ In contrast, a Mendelian randomization analysis showed that height has a positive association with risk of HNC.^11^

Importantly, however, the majority of studies on associations between anthropometric indexes and risk of HNC were conducted in Europe, and few reports have examined these associations in Asian populations.^12^ Moreover, given that smoking^13^^,^^14^ and alcohol consumption^4^ have been established as causes of HNC, any examination of associations between BMI and HNC must take account of this residual confounding using stratified analysis for these factors. Here, we investigated associations of BMI with height and HNC risk among a Japanese population stratified by smoking and alcohol consumption.

## METHODS

### Study population

The Japan Public Health Center-based Prospective Study (JPHC Study) is a cohort study that aims to explore correlations between lifestyle practices and disease occurrence. Details of the JPHC Study have been reported elsewhere.^15^ In brief, a total of 140,420 participants aged 40 to 69 years at baseline survey (68,722 men and 71,698 women) and residing in 11 public health center (PHC) areas across Japan (Iwate-Ninohe, Akita-Yokote, Nagano-Saku, Tokyo-Katsushika, Okinawa-Chubu, Niigata-Nagaoka, Ibaraki-Mito, Osaka-Suita, Kochi-Chuohigashi, Nagasaki-Kamigoto, and Okinawa-Miyako) were enrolled between 1990 and 1994. All participants were identified through population registries within the jurisdictions overseen by public health centers. For this analysis, participants from Tokyo-Katsushika (*n* = 7,097) were excluded owing to the absence of data on cancer incidence. Moreover, we further excluded participants who had non-Japanese nationality (*n* = 52), relocated their residence away from the study area before the study began (*n* = 188), were out of the age range (*n* = 7), refused follow-up (*n* = 29), had duplicated registration (*n* = 12), died or moved overseas before study commencement (*n* = 164), did not answer the questionnaire (*n* = 26,738), reported a history of cancer regardless of site (*n* = 2,227), did not report their weight or height (*n* = 1,136), or had an implausible BMI (<14 or >40; *n* = 102). Finally, 102,668 participants were analyzed in this study (eFigure 1). This study was approved by the Institutional Review Boards of the National Cancer Center (approval number: 2001-021). All participants were informed about the objectives of the study, and that completion of the survey questionnaire was considered as providing consent to participate.

### Exposure measurement

Self-administered questionnaires were distributed and collected at the baseline survey. Participants were asked to report their height and weight as at baseline. BMI was calculated by dividing weight in kilograms by the squared height in meters. The following five categories were used in previous systematic reviews examining the association of BMI and HNC^6^: <21 kg/m^2^, 21–22.9 kg/m^2^, 23–24.9 kg/m^2^, 25.0–29.9 kg/m^2^, and ≥30 kg/m^2^. However, this study used six BMI groups to determine which categories are statistically significant between BMI and HNC: <18.5 kg/m^2^, 18.5–20.9 kg/m^2^, 21.0–22.9 kg/m^2^, 23.0–24.9 kg/m^2^ (reference), 25.0–27.4 kg/m^2^, and ≥27.5 kg/m^2^. In addition, height was stratified into gender-specific quartiles (Q1–Q4), namely <160 cm, 160–<164 cm (reference), 164–<168 cm, and ≥168 cm for men; and <148 cm, 148–<152 cm (reference), 152–<156 cm, and ≥156 cm for women. Height references were set based on the average height of 50 to 59 year olds in 1990 (men: 162.8 cm, women: 151.4 cm).^16^

### Follow-up and case ascertainment

Participants were monitored from initiation of the baseline survey until they reached an endpoint, namely the date of head and neck cancer diagnosis, relocation from their originally registered area, date of death, or the conclusion of follow-up (December 31, 2013; excluding Osaka-Suita, December 31, 2012), whichever occurred first. Detection of HNC cases involved active notification of patients from large local hospitals within the study areas and linkage of records with data from population-based cancer registries. Cancer diagnosis was coded with the International Classification of Diseases for Oncology, Third Edition (C00.0–14.0).^17^ Diagnoses were classified as made using biopsy (45.5%), pathology after surgery (33.5%), cytology (1.9%), no histological diagnosis (9.0%), and unclassified (10%).

### Statistical analysis

General characteristics across different BMI and height groups were compared by analysis of variance and the chi-squared test. Hazard ratios (HRs) and 95% confidence intervals (CIs) for the association between anthropometric measurements and HNC risk were estimated using multivariate Cox proportional hazards regression. Moreover, sparse-data bias due to the small number of HNC cases was addressed by performing Firth’s bias correction.^18^^,^^19^ We used 23–24.9 kg/m^2^ for the BMI group and the second quartile for height as reference groups to calculate the effect estimates for both BMI and height exposures. Analyses were adjusted for potential confounding factors: age (continuous); sex (men, women); PHC area (ten areas); smoking status (missing, never; former; current: <20, 20–39, 40–59, and ≥60 pack years); alcohol consumption (missing, none; occasional: <1 time/week, regular: 1–149, 150–299, 300–449 g ethanol/week, heavy: ≥450 g ethanol/week); and preference for hot foods and hot drinks (missing, yes, no). Smoking, alcohol consumption, and preference for hot foods and drinks were obtained from the self-reported questionnaire at baseline. In addition to estimating between-category HRs, we also included continuous BMI and height as linear and quadratic terms. The HRs for BMI of 16.25, 19.75, 22, 26.25, and 33.75 kg/m^2^, with a reference of 24 kg/m^2^, and for height of 148, 154, and 168 cm, with a reference of 154 cm, were estimated from this quadratic association model by combining the estimated coefficients of BMI terms. For example, HR for BMI of 16.25 kg/m^2^ versus 24 kg/m^2^ was calculated as exp[(16.25 − 24)*b*_1_ + (16.25^2^ − 24^2^)*b*_2_], where *b*_1_ and *b*_2_ are adjusted estimates of the linear and quadratic term coefficients, respectively. Example SAS code is provided in eMaterial 1.

To further explore effect modification by smoking and alcohol consumption, the regression models were stratified by smoking status and alcohol consumption. To avoid potential bias arising from undiagnosed HNC, sensitivity analyses were performed, excluding HNC cases identified within the first 3 years of the study period. As a supplemental analysis, we examined the risk of HNC by site, as follows: oral cavity cancers, C01–C06; nasopharyngeal cancers, C11; oropharyngeal cancers, C09–C10; hypopharyngeal cancers, C12–C13; and salivary gland cancers, C07–C08. Different cut-off values were used to categorize BMI due to the presence of cells with zero cases, namely <18.5 kg/m^2^, 18.5–24.9 kg/m^2^ (reference), and ≥25.0 kg/m^2^, based on a past study conducted in an Asian population.^12^ All reported *P* values were two-sided, with significance established at *P* < 0.05. All statistical analyses were carried out using SAS version 9.4. software (SAS Institute, Cary, NC, USA).

## RESULTS

Over the course of 1,921,345 person-years (average 18.7; standard deviation, 6.1 years) of follow-up, a total of 311 participants (219 men and 92 women) were newly diagnosed with HNC. Of the 282 cases with specified histology, 216 cases (70%) were squamous cell carcinoma.

Table 1 shows the characteristics of participants at baseline by BMI and height. Among men, although participants with lower BMI tended to smoke, participants in the lowest category for height tended to be never-smokers. Regarding alcohol consumption, participants with lower BMI and in the lowest height category tended to be non- or occasional drinkers. Among women, we saw no evident differences by BMI in smoking or alcohol consumption. Participants with the lowest height category tended to be never-smokers and non- or occasional drinkers.

**Table 1.** Baseline characteristics of participants by BMI and height (*n* = 102,668)

	Men (*n* = 49,028)							Women (*n* = 53,640)						
	
**BMI, kg/m^2^**	<18.5	18.5–20.9	21–22.9	23–24.9	25–27.4	≥27.5	*P* value	<18.5	18.5–20.9	21–22.9	23–24.9	25–27.4	≥27.5	*P* value
Number of participants	1,358	7,929	12,710	13,667	9,285	4,079		2,027	9,936	14,136	12,839	9,364	5,338	

Age, years, mean (SD)	53.7 (9.1)	52.2 (8.4)	51.9 (8.1)	51.5 (7.8)	51.1 (7.6)	50.6 (7.3)	<0.001	52.1 (8.9)	50.5 (8.3)	51.4 (8.0)	52.1 (7.8)	53.0 (7.8)	53.1 (7.7)	<0.001
Current smokers, %	62.3	61.8	55.1	47.8	44.6	42.3	<0.001	11.4	8.2	6.1	4.7	5.3	6.6	0.16
Alcohol intake, %	58.2	65.5	67.7	67.9	65.6	62.2	<0.001	15.7	15.7	13.8	11.7	10.2	8.7	0.19
Prefer hot foods and drinks, %	24.4	24.5	23.7	22.8	23.6	22.8	0.19	34.5	33.0	32.1	32.1	31.8	28.9	0.11

**Height, cm**	Q1 (<160)	Q2 (160 to <164)	Q3 (164 to <168)	Q4 (≥168)				Q1 (<148)	Q2 (148 to <152)	Q3 (152 to <156)	Q4 (≥156)			
Number of participants	9,922	12,790	11,674	14,642				9,860	15,347	14,813	13,620			

Age, years, mean (SD)	55.4 (7.5)	52.5 (7.8)	51.1 (7.7)	48.6 (7.3)			<0.001	55.5 (7.9)	52.9 (7.9)	51.0 (7.7)	49.0 (7.4)			<0.001
Current smokers, %	45.5	50.3	51.8	55.7			<0.001	4.7	5.3	6.4	8.4			<0.001
Alcohol intake, %	61.0	64.6	66.7	71.0			<0.001	7.7	10.9	13.2	17.4			<0.001
Prefer hot foods and drinks, %	22.9	22.7	23.6	24.6			<0.001	30.6	31.2	32.4	33.4			<0.001

BMI, body mass index; SD, standard deviation.

The chi-square test was used for categorical data and analysis of variance for continuous data.

In Table 2, model 1, which was adjusted for other confounding covariates, shows that lower (<21 kg/m^2^) BMI was associated with increased risk of HNC incidence (HR 2.75; 95% CI, 1.63–4.64 for <18.5 kg/m^2^ and HR 1.63; 95% CI, 1.15–2.30 for 18.5 to 20.9 kg/m^2^). Moreover, increased risk was suggested for higher BMI, with an HR of 1.30 (95% CI, 0.84–2.00) for ≥27.5 kg/m^2^. Model 2, which included linear and quadratic terms of BMI as a continuous variable, also indicated that both lower (16.25 kg/m^2^) and higher (33.75 kg/m^2^) BMI showed increased risk of HNC incidence (HR 3.27; 95% CI, 2.04–5.23 and HR 1.72; 95% CI, 0.94–3.14, respectively). In further analysis by sex, model 1 showed that lower (<23 kg/m^2^) BMI was associated with increased risk of HNC incidence in men (<18.5 kg/m^2^: HR 3.38; 95% CI, 1.74–6.57, 18.5–20.9 kg/m^2^: HR 2.46; 95% CI, 1.62–3.71 and 21–22.9 kg/m^2^: HR 1.71; 95% CI, 1.15–2.53). Moreover, increased risk was suggested among higher BMI, with an HR of 1.29 (95% CI, 0.74–2.26) for ≥27.5 kg/m^2^. The same result was observed for the model with quadratic terms in model 2. Meanwhile, among women, increased risk of HNC was suggested only in participants having a lower BMI (<18.5 kg/m^2^: HR 1.95; 95% CI, 0.80–4.48) in model 1. The same result was observed in model 2.

**Table 2.** Hazard ratios for the association between BMI and head and neck cancer risk (*n* = 102,668)

BMI category, kg/m^2^	Midpoint, kg/m^2^	PY	Event (*n*)	Rate/100,000 PY	Model 1: BMI category			Model 2: continuous BMI (quadratic)		
	
					HR	95% CI		HR	95% CI	
<18.5	16.25	57,612.1	18	31.2	2.75	1.63	4.64	3.27	2.04	5.23
18.5–20.9	19.75	326,311.2	63	19.3	1.63	1.15	2.30	1.60	1.32	1.95
21–22.9	22	501,299.2	83	16.6	1.32	0.95	1.82	1.18	1.10	1.28
23–24.9	24	503,276.8	66	13.1	1.00	Ref		1.00	Ref	
25–27.4	26.25	354,809.9	51	14.4	1.09	0.75	1.57	0.93	0.86	1.00
≥27.5	33.75	178,206.1	30	16.8	1.30	0.84	2.00	1.72	0.94	3.14
**Men**										
<18.5	16.25	21,762.7	11	50.5	3.38	1.74	6.57	5.22	3.02	9.04
18.5–20.9	19.75	139,029.7	53	38.1	2.46	1.62	3.71	1.98	1.58	2.49
21–22.9	22	228,624.4	63	27.6	1.71	1.15	2.53	1.29	1.18	1.42
23–24.9	24	250,446.5	41	16.4	1.00	Ref		1.00	Ref	
25–27.4	26.25	170,793.2	33	19.3	1.12	0.71	1.77	0.86	0.78	0.96
≥27.5	33.75	73,992.3	18	24.3	1.29	0.74	2.26	1.58	0.73	3.39
**Women**										
18.5–20.9	16.25	35,849.4	7	19.5	1.95	0.85	4.48	1.40	0.58	3.38
21–22.9	19.75	187,281.5	10	5.3	0.58	0.28	1.21	1.09	0.76	1.57
23–24.9	22	272,674.8	20	7.3	0.75	0.42	1.36	1.01	0.88	1.17
25–27.4	24	252,830.3	25	9.9	1.00	Ref		1.00	Ref	
≥27.5	26.25	184,016.6	18	9.8	0.95	0.52	1.75	1.05	0.92	1.20
18.5–20.9	33.75	104,213.7	12	11.5	1.16	0.58	2.32	2.00	0.79	5.05

BMI, body mass index; CI, confidence interval; HR, hazard ratio; PHC, public health center; PY, person-years.

Model 1 compares the six BMI categories using dummy variables with the category “23–24.9 kg/m^2^” as a reference. The model adjusted for age (continuous); PHC area; smoking status (missing; never; former; current: <20, 20–39, 40–59, and ≥60 pack years); alcohol consumption (missing; none; <1 time/week; 1–149, 150–299, 300–449, and ≥450 g ethanol/week); hot food and drinks (missing; yes; no).

Model 2 included linear and quadratic terms of BMI as a continuous variable. The estimated coefficients were converted into HR at the midpoint value of each category compared to BMI of 24 kg/m^2^. The model adjusted for age (continuous); PHC area; smoking status (missing; never; former; current: <20, 20–39, 40–59, and ≥60 pack years); alcohol consumption (missing; none; <1 time/week; 1–149, 150–299, 300–449, and ≥450 g ethanol/week); hot food and drinks (missing; yes; no).

Table 3 shows the results of the regression model stratified by smoking status. Among never-smokers, HNC risk was increased in participants having a lower BMI (<18.5 kg/m^2^: HR 2.65; 95% CI, 1.16–6.05) and was suggested to be increased in participants having a higher BMI (≥27.5 kg/m^2^: HR 1.66; 95% CI, 0.91–3.03) in model 1. Model 2 showed a similar trend (16.25 kg/m^2^: HR 2.05; 95% CI, 0.93–4.53 and 33.75 kg/m^2^: HR 2.49; 95% CI, 1.16–5.37). After excluding HNC cases diagnosed during the first 3 years of follow-up, similar results were observed. In former and current smokers, lower BMI showed a stronger association with HNC risk, but higher BMI showed no association with HNC risk (<18.5 kg/m^2^: HR 3.09; 95% CI, 1.54–6.20 and ≥27.5 kg/m^2^: HR 1.04; 95% CI, 0.55–2.00) in model 1. In model 2, elevated risk of HNC was seen in both lower and higher BMI (16.25 kg/m^2^: HR 4.84; 95% CI, 2.65–8.84 and 33.75 kg/m^2^: HR 1.33; 95% CI, 0.52–3.44). After excluding HNC cases diagnosed during the first 3 years of follow-up, although <18.5 kg/m^2^ was not significantly associated with HNC risk (HR 1.70; 95% CI, 0.63–4.63), 18.5–20.9 and 21–22.9 kg/m^2^ were significantly associated with HNC risk (HR 2.54; 95% CI, 1.60–4.05 and HR 1.64; 95% CI, 1.04–2.59, respectively).

**Table 3.** Hazard ratios for the association between BMI and head and neck cancer risk stratified by smoking status (*n* = 100,925)

BMI category, kg/m^2^	Midpoint, kg/m^2^	PY	Event (*n*)	Rate/100,000 PY	Model 1: BMI category			Model 2: continuous BMI (quadratic)		
	
					HR	95% CI		HR	95% CI	
**Never smokers (*n* = 60,607)**										
<18.5	16.25	35,095.7	7	19.9	2.65	1.16	6.05	2.05	0.93	4.53
18.5–20.9	19.75	195,513.4	14	7.2	0.94	0.49	1.79	1.27	0.91	1.76
21–22.9	22	302,874.0	27	8.9	1.05	0.62	1.80	1.06	0.94	1.21
23–24.9	24	301,671.0	27	9.0	1.00	Ref		1.00	Ref	
25–27.4	26.25	220,050.7	23	10.5	1.13	0.65	1.98	1.03	0.92	1.16
≥27.5	33.75	118,066.5	18	15.2	1.66	0.91	3.03	2.49	1.16	5.37
**Never smokers (excluding the first three years of follow-up, *n* = 60,600)**										
<18.5	16.25	35,095.7	7	19.9	2.92	1.27	6.72	2.31	1.04	5.16
18.5–20.9	19.75	195,513.4	14	7.2	1.02	0.53	1.96	1.33	0.95	1.86
21–22.9	22	302,869.8	25	8.3	1.05	0.60	1.83	1.08	0.95	1.24
23–24.9	24	301,669.2	25	8.3	1.00	Ref		1.00	Ref	
25–27.4	26.25	220,047.3	21	9.5	1.12	0.62	1.99	1.02	0.91	1.15
≥27.5	33.75	118,064.9	17	14.4	1.70	0.91	3.16	2.58	1.19	5.58
**Former and current smokers (*n* = 40,318)**										
<18.5	16.25	21,519.4	10	46.5	3.09	1.54	6.20	4.84	2.65	8.84
18.5–20.9	19.75	126,561.3	48	37.9	2.27	1.47	3.50	1.95	1.53	2.49
21–22.9	22	190,847.8	54	28.3	1.56	1.02	2.37	1.29	1.17	1.42
23–24.9	24	192,921.5	37	19.2	1.00	Ref		1.00	Ref	
25–27.4	26.25	129,022.0	26	20.2	1.02	0.61	1.68	0.85	0.76	0.96
≥27.5	33.75	57,108.0	12	21.0	1.04	0.55	2.00	1.33	0.52	3.44
**Former and current smokers (excluding the first three years of follow-up, *n* = 40,289)**										
<18.5	16.25	21,510.9	4	18.6	1.70	0.63	4.63	3.71	1.78	7.74
18.5–20.9	19.75	126,556.2	44	34.8	2.54	1.60	4.05	1.82	1.37	2.41
21–22.9	22	190,837.6	47	24.6	1.64	1.04	2.59	1.28	1.14	1.43
23–24.9	24	192,910.7	31	16.1	1.00	Ref		1.00	Ref	
25–27.4	26.25	129,015.8	22	17.1	1.02	0.59	1.77	0.82	0.71	0.95
≥27.5	33.75	57,105.1	10	17.5	1.04	0.51	2.11	0.77	0.21	2.87

BMI, body mass index; CI, confidence interval; HR, hazard ratio; PHC, public health center; PY, person-years.

Model 1 compares the six BMI categories using dummy variables with the category “23 to 24.9” as a reference. The model adjusted for sex; age (continuous); PHC area; alcohol consumption (missing; none; <1 time/week; 1–149, 150–299, 300–449, and ≥450 g ethanol/week); hot food and drinks (missing; yes; no).

Model 2 included linear and quadratic terms of BMI as a continuous variable. The estimated coefficients were converted into HR at the midpoint value of each category compared to BMI of 24 kg/m^2^. The model adjusted for sex; age (continuous); PHC area; alcohol consumption (missing; none; <1 time/week; 1–149, 150–299, 300–449, and ≥450 g ethanol/week); hot food and drinks (missing; yes; no).

The regression model stratified by alcohol consumption is shown in Table 4. Among regular drinkers, lower BMI (<23 kg/m^2^) showed an association with increased risk of HNC incidence, and increased risk was suggested among participants having higher BMI in model 1 (HR 3.52; 95% CI, 1.53–8.09 for <18.5 kg/m^2^, HR 1.82; 95% CI, 1.04–3.19 for 18.5–20.9 kg/m^2^, HR 1.51; 95% CI, 0.90–2.53 for 21–22.9 kg/m^2^ and HR 1.65; 95% CI, 0.83–3.30 for ≥27.5 kg/m^2^). Among heavy drinkers, similar results were observed. However, among non- and occasional drinkers, point estimates in the highest category (≥27.5 kg/m^2^) showed no increase in risk (HR 1.07, 95% CI, 0.55–2.10), and elevated risk was observed only in participants having BMI <18.5 kg/m^2^ (HR 2.25; 95% CI, 1.04–4.89) in model 1.

**Table 4.** Hazard ratios for the association between BMI and head and neck cancer risk stratified by alcohol consumption (*n* = 100,590)

BMI category, kg/m^2^	Midpoint, kg/m^2^	PY	Event (*n*)	Rate/100,000 PY	Model 1: BMI category			Model 2: continuous BMI (quadratic)		
	
					HR	95% CI		HR	95% CI	
**Non and Occasional drinker (*n* = 61,358)**										
<18.5	16.25	38,240.6	8	20.9	2.25	1.04	4.89	1.97	0.90	4.33
18.5–20.9	19.75	200,584.4	17	8.5	0.91	0.50	1.67	1.28	0.93	1.77
21–22.9	22	299,499.2	25	8.3	0.86	0.50	1.47	1.08	0.95	1.23
23–24.9	24	294,629.7	29	9.8	1.00	Ref		1.00	Ref	
25–27.4	26.25	217,239.1	23	10.6	1.07	0.62	1.85	0.99	0.88	1.12
≥27.5	33.75	119,264.2	12	10.1	1.07	0.55	2.10	1.78	0.71	4.52
**Regular drinker (*n* = 33,387)**										
<18.5	16.25	15,994.9	7	43.8	3.52	1.53	8.09	4.15	1.99	8.65
18.5–20.9	19.75	103,354.3	25	24.2	1.82	1.04	3.19	1.69	1.24	2.31
21–22.9	22	164,288.3	35	21.3	1.51	0.90	2.53	1.19	1.05	1.34
23–24.9	24	169,105.7	25	14.8	1.00	Ref		1.00	Ref	
25–27.4	26.25	110,559.6	20	18.1	1.18	0.65	2.13	0.97	0.86	1.10
≥27.5	33.75	44,438.5	12	27.0	1.65	0.83	3.30	3.08	1.34	7.08
**Heavy drinker (*n* = 5,855)**										
<18.5	16.25	2,227.1	2	89.8	3.01	0.73	12.34	7.68	2.80	21.07
18.5–20.9	19.75	16,379.2	18	109.9	3.25	1.51	7.01	2.44	1.59	3.76
21–22.9	22	27,906.2	19	68.1	1.94	0.91	4.11	1.42	1.19	1.70
23–24.9	24	30,015.2	11	36.6	1.00	Ref		1.00	Ref	
25–27.4	26.25	19,970.4	6	30.0	0.75	0.28	2.00	0.78	0.64	0.94
≥27.5	33.75	10,587.8	6	56.7	1.49	0.55	4.06	1.01	0.27	3.88

BMI, body mass index; CI, confidence interval; HR, hazard ratio; PHC, public health center; PY, person-years.

Regular drinker: 1–149, 150–299, 300–449 g ethanol/week, heavy drinker: ≥450 g ethanol/week.

Model 1 compares the six BMI categories using dummy variables with the category “23 to 24.9” as a reference. The model adjusted for sex; age (continuous); PHC area; smoking status (missing; never; former; current: <20, 20–39, 40–59, and ≥60 pack years); hot food and drinks (missing; yes; no).

Model 2 included linear and quadratic terms of BMI as a continuous variable. The estimated coefficients were converted into HR at the midpoint value of each category compared to BMI of 24 kg/m^2^. The model adjusted for sex; age (continuous); PHC area; smoking status (missing; never; former; current: <20, 20–39, 40–59, and ≥60 pack years); hot food and drinks (missing; yes; no).

In contrast, we saw no statistically significant relationship between lower or higher height and risk of HNC incidence (Table 5). With regard to the association of BMI with HNC risk by site, we found no significant differences from the main analysis. (eTable 1). Similar results were obtained on analysis by smoking status (eTable 2). All models were also analyzed using only age, area, and smoking as adjustments. Results were consistent with those adjusted for all variables.

**Table 5.** Hazard ratios for the association between height and head and neck cancer risk (*n* = 102,668)

Height category, cm	Midpoint, kg/m^2^	PY	Event (*n*)	Rate/100,000 PY	Model 1: BMI category			Model 2: continuous BMI (quadratic)		
	
					HR	95% CI		HR	95% CI	
<151	148	371,118.2	64	17.2	0.79	0.57	1.08	1.02	0.86	1.20
151–156.9	154	532,450.7	95	17.8	1.00	Ref		1.00	Ref	
157–163.9	160.5	499,269.8	78	15.6	0.98	0.72	1.32	1.03	0.90	1.17
≥164	168	518,676.6	74	14.3	0.94	0.69	1.28	1.13	0.87	1.47
**Men**										
<160	156	178,738.0	44	24.6	0.73	0.50	1.08	1.01	0.89	1.15
160–163.9	162	233,298.6	65	27.9	1.00	Ref		1.00	Ref	
164–167.9	166	211,993.8	55	25.9	1.03	0.72	1.48	1.05	0.97	1.14
≥168	170	260,618.6	55	21.1	0.97	0.67	1.40	1.16	0.98	1.38
**Women**										
<148	145	192,380.2	20	10.4	0.93	0.53	1.66	0.95	0.75	1.22
148–151.9	150	299,152.2	30	10.0	1.00	Ref		1.00	Ref	
152–155.9	154	287,276.0	23	8.0	0.90	0.52	1.55	1.03	0.88	1.20
≥156	158	258,058.0	19	7.4	0.91	0.51	1.64	1.05	0.75	1.46

BMI, body mass index; CI, confidence interval; HR, hazard ratio; PHC, public health center; PY, person-years.

Model 1 compares the four height categories using dummy variables with the category “151 to 156.9” as a reference. The model adjusted for age (continuous); PHC area; smoking status (missing; never; former; current: <20, 20–39, 40–59, and ≥60 pack years); alcohol consumption (missing; none; <1 time/week; 1–149, 150–299, 300–449, and ≥450 g ethanol/week); hot food and drinks (missing; yes; no).

Model 2 included linear and quadratic terms of height as a continuous variable. The estimated coefficients were converted into HR at the midpoint value of each category compared to height of 154 cm. The model adjusted for age (continuous); PHC area; smoking status (missing; never; former; current: <20, 20–39, 40–59, and ≥60 pack years); alcohol consumption (missing; none; <1 time/week; 1–149, 150–299, 300–449, and ≥450 g ethanol/week); hot food and drinks (missing; yes; no).

## DISCUSSION

In this study of associations between BMI, height, and incidence of HNC, we found that the results for five categories (model 1) suggest a U-shaped change in hazard. Moreover, HRs estimated using model 2, which included this U-shaped change, were consistent with these model 1 results. These findings therefore indicate a U-shaped association of BMI with HNC risk, even after accounting for smoking and other potential influencing factors. Moreover, this U-shaped association was also observed among never-smokers. Smoking as well as alcohol are both reported to be important risk factors of HNC,^13^ and should, therefore, be considered when exploring the association of BMI with risk of HNC. Meanwhile, we saw no statistically significant association between height and HNC risk.

Regarding never-smokers, several studies have reported that both lower and higher BMI are associated with increased risk of HNC.^6^^,^^12^ In particular, a pooled analysis involving 20 cohort studies encompassing 1.9 million subjects revealed that a BMI level below 21 or above 30 kg/m^2^ was associated with heightened HNC risk among never-smokers (HR 1.17; 95% CI, 0.85–1.61 and HR 1.40; 95% CI, 1.08–1.81, respectively). In Asia, Chen et al also found that HNC risk was increased at both <18.5 kg/m^2^ (odds ratio [OR] 2.18; 95% CI, 0.97–4.87) and ≥30 kg/m^2^ (OR 1.37; 95% CI, 0.55–3.42) among never-smokers.^12^ Our results support these previous studies.

Meanwhile, our present study also showed that only lower BMI (<23 kg/m^2^) was associated with HNC risk in both former and current smokers. Several studies reported that lower BMI showed an association with HNC risk, while higher BMI was not associated with this risk.^5^^,^^6^^,^^12^^,^^20^ In the pooled analysis involving 1.9 million participants across 20 cohort studies, while lower BMI (<21 kg/m^2^) showed an association with heightened risk of HNC, there was no evidence suggesting that increased risk was associated with higher BMI (≥30 kg/m^2^) among either former or current smokers (HR 0.96; 95% CI, 0.79–1.18 and HR 0.58; 95% CI, 0.47–0.72, respectively).^6^ In Asia, Chen et al also noted that although lower BMI (<18.5 kg/m^2^) showed an association with increased risk of HNC, point estimates of higher BMI (≥30 kg/m^2^) did not show increased risk in ever-smokers (OR 0.10; 95% CI, 0.04–0.24).^12^ Moreover, a pooled analysis involving 17 case-control studies showed that while BMI <18.5 kg/m^2^ showed an association with increased HNC risk among ever-smokers (OR 2.17; 95% CI, 1.78–2.64), point estimates of higher BMI again did not show increased risk among ever-smokers (OR 0.41; 95% CI, 0.32–0.51).^5^ Although we observed a non-linear trend among former as well as current smokers in this study, this trend disappeared after we excluded HNC cases diagnosed within the first 3 years of the study period. Moreover, higher BMI showed no association with HNC risk among former and current smokers. The WCRF pointed out that weight loss may occur early in the disease course among smokers.^4^ Participants might, therefore, have already experienced weight loss and other symptoms due to HNC at the time of questionnaire response. Future studies to confirm these results are required.

With regard to stratification by alcohol consumption, a hospital-based multicenter case-control study found that only lower BMI showed a significant association with risk of HNC in both never and ever-drinkers.^12^ A large multicenter population-based case-control study reported that the association was observed only with low BMI among ever-drinkers and that—although without statistical significance—HNC risk was increased with BMI <18.5 kg/m^2^ among never-drinkers (OR 3.83; 95% CI, 0.69–21.28).^20^ Similar to previous studies, our present study showed an increased HNC risk also among participants with BMI <18.5 kg/m^2^ in both non- and occasional drinkers, regardless of alcohol consumption. However, in contrast to previous studies, we also showed an increased HNC risk in higher BMI among regular drinkers. Although the reason is not clear, Chen et al reported that HNC risk tended to be increased with BMI ≥30 kg/m^2^ at age 20 among ever-drinkers (OR 1.58; 95% CI, 0.22–11.57) in a Chinese population.^12^ Higher BMI status over a long period of time may affect HNC risk.

Although the mechanism of the increased risk of HNC with lower BMI has not been established, the association of smoking with lower BMI^21^ and its strong impact on HNC incidence^13^ both indicate that the association is strongly confounded by smoking status. A positive association with lower BMI has also been noted in lung cancer,^22^ and some reports have proposed that BMI is negatively correlated with levels of benzo(a)pyrene adduct as well as urinary 8-hydroxydeoxyguanosine, both of which are implicated in tobacco-related carcinogenesis.^23^^,^^24^ The same reasoning may be considered for HNC, which is strongly related to smoking. In the present study, however, we identified an association between lower BMI and increased HNC risk among never-smokers. In Japan, a U-shaped association of BMI with development of cancer has been seen among men.^3^ Further research on the biological mechanisms underlying these findings is needed.

Meanwhile, we also found that increased HNC risk in higher BMI among never-smokers in this study. Obesity has been noted as a possible contributor to tumorigenesis.^21^ For example, hyperadiposity has been shown to have an association with altered function of adipose tissue, as well as adipocyte death, and chronic low-grade inflammation, together with an elevated risk of both the development and progression of cancer.^25^ Further investigation to elucidate the association of obesity with HNC risk is warranted.

In this study, we observed a U-shaped association between BMI and HNC risk. While this increased risk of HNC in the low BMI range, derived from a large population, carries significant public health implications, population size was substantially decreased in the high BMI range, particularly around BMI 35 kg/m^2^, where risk escalates. Moreover, the heightened risk of HNC appeared to be limited at BMI up to approximately 30 kg/m^2^.

In this study, height showed no association with HNC risk. Previous findings regarding the relationship between height and HNC risk have been inconclusive. For instance, a meta-analysis of 20 cohort studies, including two studies from Asia, indicated that taller stature was associated with higher HNC risk^6^; another pooled analysis of 24 case-control studies, also including two studies from Asia, reported an inverse association between height and HNC risk^10^; while a large prospective cohort study in women reported that mouth and pharynx cancer were not associated with height.^26^ These studies were primarily obtained from subjects in Europe and the United States; further research in Asia will allow these differences to be examined by region.

There are several limitations in this study. Anthropometric measurements were sourced using a self-completed questionnaire at baseline. Measurement error might, therefore, have affected the results. However, the accuracy of height and weight data in the JPHC Study has been confirmed through validation against data from health check-ups, yielding Spearman correlation coefficients for BMI in men and women of 0.89 and 0.91, respectively.^27^ In this study, smoking and alcohol consumption were not treated as time-varying variables. Therefore, it is important to consider that these results reflect only the smoking and alcohol consumption status at baseline. Stratified analyses of smoking and alcohol consumption by sex were not performed owing to the small number of cases. In particular, we saw few HNC cases in women (men: 219 cases, women: 91 cases). A closely detailed evaluation of the association between BMI and HNC risk by amount of smoking and alcohol consumption in men and women would require a large pooled analysis.

Allowing for these limitations, this study represents the first population-based prospective cohort investigation of the association between anthropometric measurements and HNC incidence in a Japanese population. Moreover, the JPHC cohort study has several important strengths, such as a good response rate, extended follow-up period, and a prospective design.

In conclusion, lower BMI showed a significant association with HNC risk without regard to smoking status, while increased HNC risk was suggested with higher BMI among never-smokers.

## SUPPLEMENTARY MATERIAL

eTable 1. Hazard ratios for the association between BMI and head and neck cancer risk by site (n = 102,668)

eTable 2. Hazard ratios for the association between BMI and site-specific HNC risk by smoking status (n = 100,925)

eMaterial 1. SAS codes for estimating the adjusted hazard ratios from the fitted Cox models with dummy variables for categorized BMI (model 1) and continuous BMI included as linear and quadratic terms (model 2)

eFigure 1. Flowchart of the study
