# Supplementary material for: Body Mass Index, Height, and Head and Neck Cancer Risk: The Japan Public Health Center-based Prospective Study
Source: J Epidemiol. 2025 Apr 5;35(4):170–7. doi: 10.2188/jea.JE20240033 (PMC11882347; doi:10.2188/jea.JE20240033)
Supplement: Supplementary file 1 [file je-35-170-s001.pdf]

eMaterial 1. SAS codes for estimating the adjusted hazard ratios from the fitted Cox models with dummy variables for categorized BMI (model 1) and continuous BMI included as linear and quadratic terms (model 2)

```
* Model 1: 5-category model;
proc phreg data = oral_CHTab4;
  strata PHC;
  model ITIME * opcancer(0) = BMI1-BMI5 AGE SEX SMK1-SMK6 ALC1-ALC6 HOT1-HOT3/ rl firth;
run;

* Model 2: continuous-quadratic model;
proc phreg data = oral_CHTab4;
  strata PHC;
  model ITIME * opcancer(0) =BMI BMI*BMI AGE SEX SMK1-SMK6 ALC1-ALC6 HOT1-HOT3/ rl firth;
  estimate "BMI 16.25 v 24" bmi %sysevalf(16.25 - 24) bmi*bmi  %sysevalf(16.25**2 - 24**2) / exp cl;
  estimate "BMI 19.75 v 24" bmi %sysevalf(19.75 - 24) bmi*bmi  %sysevalf(19.75**2 - 24**2) / exp cl;
  estimate "BMI 22 v 24" bmi %sysevalf(22 - 24) bmi*bmi  %sysevalf(22**2 - 24**2) / exp cl;
  estimate "BMI 26.25 v 24" bmi %sysevalf(26.25 - 24) bmi*bmi  %sysevalf(26.25**2 - 24**2) / exp cl;
  estimate "BMI 33.75 v 24" bmi %sysevalf(33.75 - 24) bmi*bmi  %sysevalf(33.75**2 - 24**2) / exp cl;
run;
```

eTable 1. Hazard ratios for the association between BMI and head and neck cancer risk by site (n=102,668)

BMI category, kg/m^2	Midpoint, kg/m^2	PY	Event (n)	Rate/100,000 PY	Model 1: BMI category HR	Model 1: 95% CI		Model 2: continuous BMI (quadratic) HR	Model 2: 95% CI	
**Oral cavity cancer**										
<23.0	18.5	885222.5	76	8.6	1.21	0.81	1.80	1.87	1.26	2.76
23–24.9	24	503276.8	36	7.2	1.00	Ref		1.00	Ref	
≥25.0	32.5	533016.0	40	7.5	1.04	0.67	1.64	1.55	0.81	2.94
**Nasopharyngeal cancers**										
<23.0	18.5	885222.5	5	0.6	1.95	0.29	13.04	1.52	0.45	5.20
23–24.9	24	503276.8	1	0.2	1.00	Ref		1.00	Ref	
≥25.0	32.5	533016.0	8	1.5	5.39	0.87	33.38	5.97	2.35	15.18
**Oropharyngeal cancers**										
<23.0	18.5	885222.5	35	4.0	1.63	0.85	3.15	2.86	1.58	5.17
23–24.9	24	503276.8	12	2.4	1.00	Ref		1.00	Ref	
≥25.0	32.5	533016.0	13	2.4	1.12	0.51	2.45	1.45	0.47	4.47
**Hypopharyngeal cancers**										
<23.0	18.5	885222.5	30	3.4	1.94	0.97	3.89	1.81	0.85	3.88
23–24.9	24	503276.8	11	2.2	1.00	Ref		1.00	Ref	
≥25.0	32.5	533016.0	14	2.6	1.07	0.49	2.37	0.80	0.18	3.45
**Salivary gland cancers**										
<23.0	18.5	885222.5	13	1.5	1.29	0.49	3.39	1.30	0.45	3.72
23–24.9	24	503276.8	6	1.2	1.00	Ref		1.00	Ref	

≥25.0	32.5	533016.0	8	1.5	1.24	0.43	3.55	2.19	0.55	8.80

BMI, body mass index; CI, confidence interval; HR, hazard ratio; PHC, public health center; PY, person-years.

Multivariate analyses were adjusted for sex; age (continuous); PHC area; smoking status (missing; never; former; current: <20, 20–39, 40–59, and ≥60 pack years); alcohol consumption (missing; none; <1 time/week; 1–149,150–299, 300–449, and ≥450 g ethanol/week); hot food and drinks (missing; yes; no).

Oral cavity cancers: C01–C06, nasopharyngeal cancers: C11, oropharyngeal cancers: C09–C10, hypopharyngeal cancers: C12–C13, salivary gland cancers: C07–C08.

eTable 2. Hazard ratios for the association between BMI and site-specific HNC risk by smoking status (n=100,925)

					Model 1: BMI category			Model 2: continuous BMI (quadratic)		
BMI category, kg/m^2	Midpoint, kg/m^2	PY	Event (n)	Rate/100,000 PY	HR	95% CI		HR	95% CI	
**Never smokers (n=60,607)**										
**Oral cavity cancer**										
<23.0	18.5	533483.1	24	4.5	0.66	0.36	1.18	1.15	0.60	2.21
23–24.9	24	301671.0	21	7.0	1.00	Ref		1.00	Ref	
≥25.0	32.5	338117.2	22	6.5	0.92	0.51	1.68	1.78	0.75	4.23
**Nasopharyngeal cancers**										
<23.0	18.5	533483.1	4	0.7	—	—	—	—	—	—
23–24.9	24	301671.0	0	0.0	1.00	Ref		1.00	Ref	
≥25.0	32.5	338117.2	5	1.5	—	—	—	—	—	—
**Oropharyngeal cancers**										
<23.0	18.5	533483.1	5	0.9	1.03	0.25	4.30	1.06	0.21	5.37
23–24.9	24	301671.0	3	1.0	1.00	Ref		1.00	Ref	
≥25.0	32.5	338117.2	4	1.2	1.16	0.27	5.12	3.85	0.85	17.48
**Hypopharyngeal cancers**										
<23.0	18.5	533483.1	6	1.1	3.56	0.54	23.66	1.54	0.31	7.75
23–24.9	24	301671.0	1	0.3	1.00	Ref		1.00	Ref	
≥25.0	32.5	338117.2	5	1.5	2.78	0.41	18.97	2.42	0.46	12.60
**Salivary gland cancers**										
<23.0	18.5	533483.1	6	1.1	1.00	0.28	3.55	1.75	0.49	6.24

23–24.9	24	301671.0	4	1.3	1.00	Ref		1.00	Ref	
≥25.0	32.5	338117.2	6	1.8	1.32	0.37	4.66	3.65	1.13	11.75
**Former and current smokers (n=40,319)**										
**Oral cavity cancer**										
<23.0	18.5	338928.5	50	14.8	1.94	1.09	3.46	2.87	1.74	4.72
23–24.9	24	192921.5	15	7.8	1.00	Ref		1.00	Ref	
≥25.0	32.5	186130.0	17	9.1	1.13	0.56	2.25	1.58	0.64	3.89
**Nasopharyngeal cancers**										
<23.0	18.5	338928.5	1	0.3	0.60	0.04	7.99	0.89	0.09	9.08
23–24.9	24	192921.5	1	0.5	1.00	Ref		1.00	Ref	
≥25.0	32.5	186130.0	3	1.6	2.24	0.25	20.37	9.72	2.26	41.74
**Oropharyngeal cancers**										
<23.0	18.5	338928.5	30	8.9	2.16	1.00	4.71	4.16	2.24	7.73
23–24.9	24	192921.5	8	4.1	1.00	Ref		1.00	Ref	
≥25.0	32.5	186130.0	9	4.8	1.20	0.47	3.10	1.34	0.34	5.24
**Hypopharyngeal cancers**										
<23.0	18.5	338928.5	23	6.8	1.79	0.82	3.89	2.17	0.91	5.15
23–24.9	24	192921.5	9	4.7	1.00	Ref		1.00	Ref	
≥25.0	32.5	186130.0	8	4.3	0.80	0.31	2.07	0.69	0.10	4.69
**Salivary gland cancers**										
<23.0	18.5	338928.5	6	1.8	1.50	0.31	7.27	0.56	0.05	6.54
23–24.9	24	192921.5	2	1.0	1.00	Ref		1.00	Ref	

≥25.0	32.5	186130.0	2	1.1	0.98	0.15	6.58	0.10	0.00	70.37

BMI, body mass index; CI, confidence interval; HR, hazard ratio; PHC, public health center; PY, person-years.

Multivariate analyses were adjusted for sex; age (continuous); PHC area; smoking status (missing; never; former; current: <20, 20–39, 40–59, and ≥60 pack years); alcohol consumption (missing; none; <1 time/week; 1–149,150–299, 300–449, and ≥450 g ethanol/week); hot food and drinks (missing; yes; no).

Oral cavity cancers: C01–C06, nasopharyngeal cancers: C11, oropharyngeal cancers: C09–C10, hypopharyngeal cancers: C12–C13, salivary gland cancers: C07–C08.

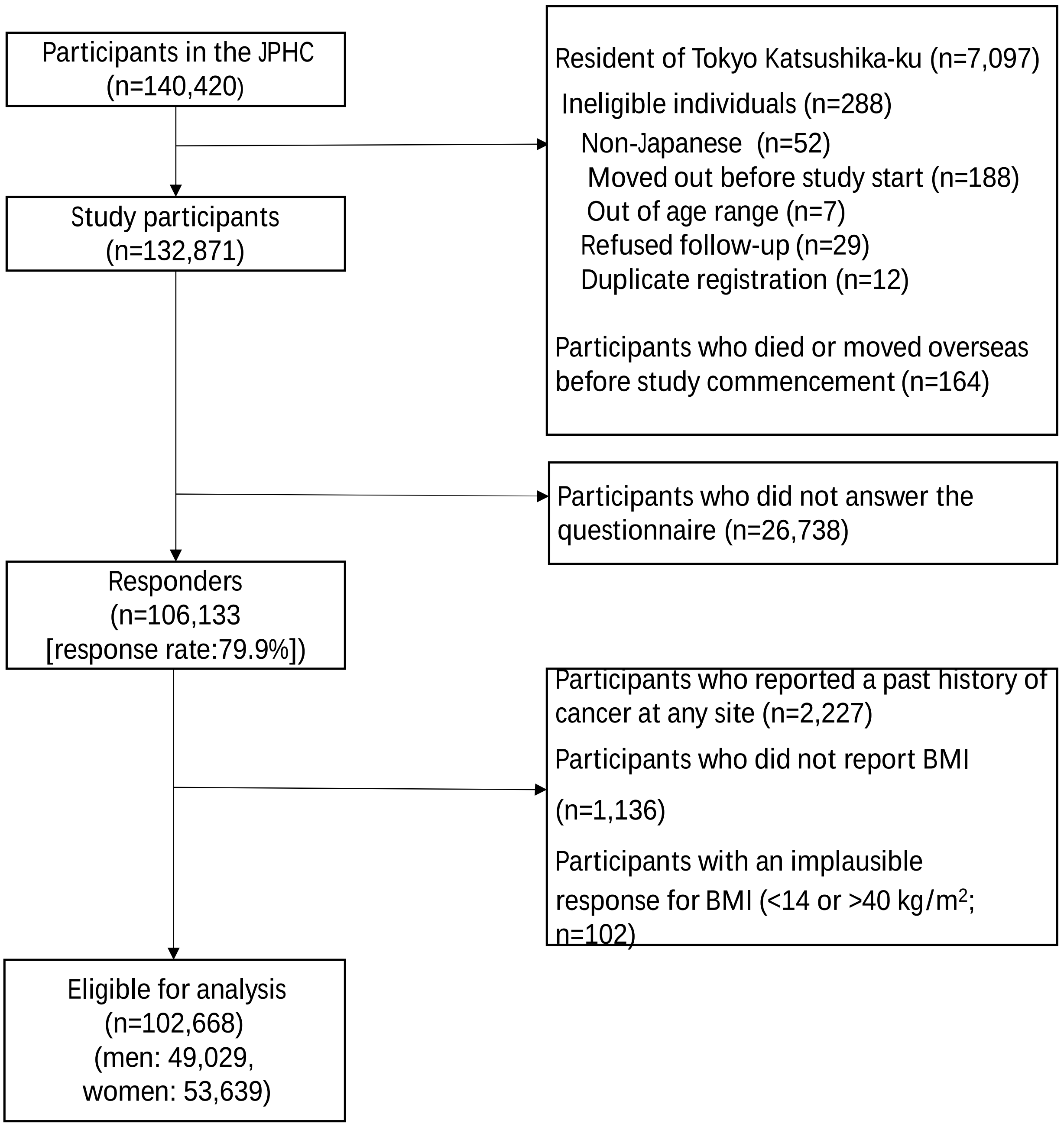

eFigure 1. Flowchart of the study
